# Current Clinical Utility of Gene Expression Panels in Primary Cutaneous Melanoma

**DOI:** 10.3390/cancers18111830

**Published:** 2026-06-03

**Authors:** Taylor L. Garza, Jae Hwan Choi, Michelle McGee, Edmond Box, Timothy Nywening, Andreas Karachristos, Abraham Schwarzberg, Mayer Fishman, Richard Jacobson

**Affiliations:** 1 Department of Surgery, University of South Florida, Tampa, FL 33606, USAjaehwanchoi@usf.edu (J.H.C.);; 2 Cancer Institute, Tampa General Hospital, Tampa, FL 33606, USA

**Keywords:** melanoma, gene expression panel, sentinel lymph node biopsy

## Abstract

Roughly 100,000 new cases of melanoma are diagnosed yearly in the United States, the majority of which are early-stage disease with excellent prognosis. However, a subset of patients harbors clinically occult aggressive biology that can go undetected on standard clinicopathologic analyses. Gene expression profiling (GEP) assays have emerged as molecular adjuncts to risk stratification, aiming to improve sentinel lymph node biopsy (SLNB) decision-making and surveillance planning. Two commercial tests are widely available: the 31-gene expression profile (31-GEP, DecisionDx-Melanoma, Castle Biosciences) and the clinicopathologic gene expression profile (CP-GEP, Merlin, SkylineDx). This review summarizes the current evidence for each assay regarding their performance and utility, with a focus on potentially actionable use cases, limitations, and the practical context in which these tools are most valuable.

## Simple Summary

This article summarizes the translational and clinical data supporting the use of two commercially available gene expression profile (GEP) assays, 31-gene expression profile (31-GEP) and clinicopathologic gene expression profile (CP-GEP), in primary cutaneous melanoma (CM). These panels are in widespread clinical practice, however data supporting their use are limited in some areas. Thus far, the areas with the best supporting data are in “high risk” clinical stage T1a melanoma, where GEP can reclassify a meaningful proportion into actionable risk categories. Ongoing investigation is facilitating their incorporation into formal clinical guidelines.

## 1. Introduction

Roughly 100,000 new cases of melanoma are diagnosed yearly in the United States, with approximately 1.5 million people living with the disease as of 2023 [1]. Incidence has risen over the past four decades, while age-adjusted mortality has remained stable [2]. The rise in incidence has been driven mainly by detection of early-stage disease. While newly diagnosed early-stage melanoma has an excellent melanoma-specific survival (MSS), a minority within this group harbor aggressive biology not captured by current clinicopathologic staging. Given the high prevalence of early stage-melanoma at diagnosis, advances in current staging techniques are warranted to improve survival within this population.

Survival decreases substantially with advancing stage. Outcomes in late-stage disease have improved meaningfully with immune and targeted therapy; thus, identifying a subset of early-stage patients with biologically aggressive disease directly impacts management. In this regard, sentinel lymph node biopsy (SLNB) has been a critical prognostic factor over the past 30 years. Prior work demonstrated that radiographic staging of early-stage disease had inadequate sensitivity to detect low volume nodal disease [3]. Thus, more recent work has focused on gene expression in the primary tumor and circulating tumor DNA (ctDNA). Specifically, that such noninvasive studies may guide surveillance, adjuvant therapeutics and the use of invasive staging including SLNB.

While SLNB remains the gold standard for nodal staging, its widespread use is associated with considerable healthcare costs. The addition of SLNB to wide excision typically requires an operating room and general anesthesia, contributing significantly to overall expenditure, particularly when weighed against its diagnostic yield in early-stage disease. In a recent population-based registry analysis examining the use and costs of SLNB in non-ulcerated T1b melanoma, only 4.1% (156 of 3835) of patients who underwent SLNB demonstrated a positive result. When analyzing the healthcare costs associated with this procedure, the national average Medicare reimbursement per patient for a wide excision with SLNB was estimated to be approximately $3200 compared to $400 for a wide excision alone [4]. SLNB also incurs a risk of wound complications ranging from simple seroma to lymphedema.

Gene expression profiles (GEP) are marketed as an adjunct to clinicopathologic staging designed to improve risk stratification. GEP are real-time polymerase chain reaction (RT-PCR)-based assays performed on shave biopsy specimens. They measure gene expression through messenger RNA quantitation. Turnaround time is typically within 2 weeks, allowing for consideration of their results in surgical planning. The tests are commonly covered by insurance or the manufacturer; however, in some cases, patients pay out of pocket [5].

Two commercial GEP are widely available: a 31-gene expression panel (31-GEP, DecisionDx-Melanoma, Castle Biosciences, Friendswood, TX, USA) and the clinicopathologic gene expression profile (CP-GEP, Merlin, SkylineDx, Rotterdam, The Netherlands) (Table 1). GEP are mentioned in the footnotes of the National Comprehensive Cancer Network (NCCN) guidelines for CM as potentially helpful in risk stratification and selection for SLNB [6]. However, neither has been formally incorporated into the guidelines due to a lack of high-level evidence [7]. GEP are nonetheless ordered by dermatologists, oncologists or surgeons without a clearly defined role in the melanoma care pathway. This review summarizes current evidence for each assay, with a focus on the underlying biology, actionable use cases, limitations and future directions.

## 2. Technical Parameters of 31-GEP and CP-GEP

The 31-GEP is a RT-PCR assay that measures the expression of 31 genes in formalin-fixed, paraffin-embedded tissue from the primary tumor. Messenger RNA transcripts of 28 discriminating genes are quantified and compared to 3 control genes. Gene selection was based on analysis of publicly available gene expression datasets to identify expression patterns associated with recurrence and distant metastases [8]. Initial validation studies published in 2018 [9] demonstrated a technical success rate of over 98%, and high inter-assay reliability on sequentially tested samples.

A subsequent model algorithmically integrated clinicopathologic features (Breslow thickness, ulceration, mitotic rate and age) with gene expression profiling in a new assay, the i31-GEP. The i31-GEP offers patients a personalized risk of recurrence and sentinel lymph node positivity. The current version of the test generates a continuous numeric risk score between 0 and 1 and then bins patients into one of four classes based on that score: Class 1A (0–0.41), Class 1B (0.42–0.49), Class 2A (0.50–0.58), and Class 2B (0.59–1.00). Higher numeric risk score and class indicate a higher risk of melanoma recurrence.

The CP-GEP test similarly integrates patient age and Breslow thickness with RT-PCR targeting 8 genes, also on formalin-fixed, paraffin-embedded tissue [10,11]. This test bins patients into one of two groups: low risk or high risk for nodal metastases. Technical success rate in a randomized controlled trial setting was 98% [12].

## 3. Biological Programs Captured by GEP Assays

Gene expression measured in the 31-GEP and CP-GEP overlaps with transcriptional programs that define melanoma cell state and tumor microenvironment (TME) biology. Melanoma cells are under transcriptional control of a MITF-rheostat among other pathways, reversibly switching between proliferative and invasive states [13]. As it applies to risk stratification, tumors with a transcriptional state on the invasive end of the spectrum at diagnosis are biologically primed for metastasis in a manner not captured by Breslow depth or ulceration. In the TME, extracellular matrix remodeling programs contribute to invasive behavior and metastatic seeding through collagenolysis and altered immune cell trafficking [14]. Other programs such as stress adaptation [15], ion channel [16,17], and androgen receptor (AR) signaling [18] are not currently captured by commercial GEP and represent an opportunity for refinement in future assays. In addition, as these are bulk tissue assays, the localization of expressed programs to one compartment or another of the TME is not possible. The practical implication is that a high risk GEP likely represents a composite of tumor-intrinsic, stromal and immune factors, and that room for future improvement remains.

## 4. Prediction of SLNB Positivity

The 8th edition of the American Joint Committee on Cancer (AJCC) staging manual for melanoma categorizes all lesions with a Breslow thickness < 0.8 mm and without ulceration as a clinical stage T1a. Lesions with 0.8 mm Breslow and above, and all ulcerated lesions, are categorized as T1b or above [19]. The bluntest use of these guidelines dictates offering SLNB for patients with T1b and above primary lesions and not offering it to patients with T1a primary lesions. This reduces the decision to offer SLNB to two primary clinicopathologic factors: Breslow depth and ulceration status. However, this paradigm may miss patients at risk for SLN positivity based on additional high risk clinicopathologic features. The footnotes of the NCCN guidelines mention patients with Breslow depth > 0.5 mm and age < 42, head/neck location, lymphovascular invasion or mitotic rate of 2/mm^2^ or greater. These additional factors define “high risk” T1a lesions that may be appropriate for SLNB, as supported by a cohort study of 965 patients with T1a primary tumors who underwent SLNB [20]. Additionally, patients with suspected sampling error on their initial biopsy based on residual pigment or a lesion transected at the base should be considered high-risk T1a.

In theory, a perfect GEP could eliminate the need for invasive staging with SLNB. In practice, current assays are marginal improvements over current risk stratification paradigms. According to the 2026 NCCN guidelines for melanoma, SLNB is not recommended for patients with a risk of SLN positivity less than 5%. It should be discussed and considered in patients with a 5–10% risk of positivity, and offered to patients with higher than 10% risk of positivity. The i31-GEP report combines clinicopathologic and 31-GEP data to produce a patient-specific risk of SLNB positivity that can be compared to these recommendations. In a multicenter prospective study of 1421 subjects, the 31-GEP strongly predicted SLNB negativity [21]. Class 1A patients over age 55 had a 4.9% SLN positivity rate, and those over 65 had a 1.6% rate, both below the established 5% threshold for even discussing SLNB. Class 2B patients had a 30.8% (age ≥ 55) and 11.9% (age ≥ 65) positivity rate. The clinically useful population is the ambiguous group with 5–10% predicted clinicopathologic SLN positivity. Among 563 such patients, application of i31-GEP reclassified 63% into an actionable category (either < 5%, where SLNB is not recommended, or >10%, where it is) [22].

A prospective, multicenter analysis compared rates of SLNB performed utilizing the i31-GEP as part of the shared decision-making process. Participants from the DECIDE study in which i31-GEP was used as an adjunct in the decision-making process were compared to a propensity matched cohort where the assay was not used. This study found that incorporating i31-GEP led to a significantly lower reduction (18.5%) in the number of SLNB performed compared to clinicopathologic variables alone [23].

The MERLIN_001 trial is the largest prospective evaluation of a melanoma GEP. The trial enrolled 1761 patients between 2021 and 2024 with T1–T3 tumors, including high-risk T1a lesions [12]. All patients underwent SLNB based on standard criteria, with blinded CP-GEP testing in parallel. Thirty-seven percent were stratified as low-risk and 63% as high-risk; low-risk patients had a 7.1% SLN positivity rate versus 23.8% in the high-risk group (negative predictive value 92.9%). Among the 21 T1a patients with low-risk CP-GEP results (80% of T1a patients in the trial), zero SLN positivity was observed even in the presence of adverse clinical features. MERLIN_001 supports the idea that CP-GEP outperforms clinicopathologic features alone for predicting SLN positivity in this population. However, a low-risk result in a patient with adverse clinicopathologic features should not, on current evidence, be used to forgo SLNB outside a clinical trial, given the absence of randomized data and the residual ~5–7% SLN positivity in CP-GEP low-risk subjects.

Counseling high-risk T1a patients on SLNB can also be supported using nomograms incorporating the clinicopathologic factors above [20,21]. The 31-GEP and CP-GEP exceed raw clinicopathologic performance, but whether they outperform current nomograms in head-to-head comparison is unsettled. Retrospective data suggest GEP outperform commonly used nomograms [24,25]; however, prospective, independent comparative validation studies are needed.

In day-to-day decision-making, three clinical scenarios illustrate where current evidence supports practical application of GEP. First, a 70-year-old with a 0.6 mm non-ulcerated T1a melanoma without high-risk features falls below the 5% SLNB threshold on standard staging; GEP is unlikely to change management and is generally not indicated. Second, a 50-year-old with a 0.6 mm T1a melanoma showing mitoses, lymphovascular invasion, or head/neck location lies in the 5–10% ambiguous range, where SLNB is discussed but not mandated; this is the scenario in which GEP has the strongest evidence-based utility, as i31-GEP can reclassify the majority of such patients into actionable <5% or >10% categories [22] and low-risk CP-GEP identifies a population with very low SLN positivity even in the presence of adverse features [12]. Third, a patient with a T2 or higher lesion is already a clear SLNB candidate. GEP can refine recurrence-risk counseling and inform surveillance intensity but should not be used to forgo SLNB outside a clinical trial. Table 2 summarizes these scenarios alongside their evidence base.

## 5. Prognostication and Surveillance

In addition to its limitations in guiding SLNB decisions, AJCC staging has also been criticized for its inability to provide sufficient individualized prognostic information. In a recent study comparing 31-GEP to standard AJCC8 staging for patients with melanoma, 31-GEP was found to significantly improve risk stratification and survival prediction for patients with stage I disease compared to current AJCC staging alone. By providing a more accurate distinction between low- (Class 1A) and high-risk (Class 2B) groups, they found that Class 1A had higher 5-year recurrence-free survival (RFS) (97.3%) compared to Class 1B/2A (88.6%) or Class 2B (77.3%). 31-GEP also demonstrated superior 5-year MSS stratification compared to standard AJCC staging with lower MSS for Class 2B (88.8%) compared to Class 1A (99.7%) and 1B/2A (97.6%) as opposed to only minor differences between AJCC Stages IA (99.5%) and IB (97.2%) [29]. There is clearly a need for more accurate and personalized risk stratification for these patients for both decision-making and prognostic benefit, and the integration of GEP assays alongside current staging guidelines may help solve this in the future.

Both 31-GEP and CP-GEP provide prognostic information beyond prediction of SLNB positivity, including recurrence, distant metastases, and MSS. An independent multicenter retrospective study evaluating the prognostic accuracy of 31-GEP found that among 523 patients, the Class 2 GEP result demonstrated a higher sensitivity for detecting recurrence, distant metastasis, and MSS (70%, 75%, and 85%, respectively) than SLN positivity (66%, 67%, and 79%, respectively). 31-GEP proved to be an independent predictor for RFS and distant metastasis-free survival (DMFS) across both univariate and multivariate analyses [30]. This prognostic utility was similarly demonstrated with the i31-GEP. A multicenter, retrospective study in 2022 evaluated whether it could improve personalized risk stratification and prognostic assessments beyond standard staging patients with Stage I-III disease. The investigators found that low-risk i31-ROR subjects demonstrated significantly higher 5-year RFS, DMFS, and MSS (91%, 95%, 98%, respectively) compared to high-risk i31-ROR subjects (45%, 53%, and 73%, respectively) [27].

The translation of prognostic accuracy into survival benefit or management change remains unproven. A SEER registry study propensity matched 4687 stage I–III patients who were tested with the 31-GEP to 9774 non-GEP tested patients. They found an association between GEP-tested patients and lower mortality rates than non-tested patients; however, prospective validation studies are needed to establish a causal relationship between the two [28]. Limitations of this study include ordering of a GEP as a confounding variable associated with higher-resource practices and more engaged patients, and no data are available on whether the test results actually changed management.

The question of how GEP assays could shape management is further complicated by evolving treatment modalities. Recent data from the CheckMate 76K trial supported the use of adjuvant checkpoint inhibition in high-risk Stage II patients, leading to the approval of Nivolumab in patients with completely resected disease [31]. While GEP-based risk stratification may help identify patients most likely to benefit from such therapy, there are no studies to date measuring the ability of these assays to predict treatment response or benefit from adjuvant therapy. Specifically, no peer-reviewed analyses to date have correlated 31-GEP or CP-GEP class with PD-L1 expression, tumor mutational burden or response to adjuvant pembrolizumab or nivolumab in melanoma.

## 6. Current Limitations of Evidence in GEP

A balanced appraisal of GEP requires attention to who has generated the evidence. A substantial fraction of the published validation literature for both 31-GEP and CP-GEP has been authored or co-authored by personnel affiliated with the assay manufacturers. Independent investigator-initiated validation, most notably the Dutch CP-GEP cohort [26] has reported more modest performance than manufacturer-led series.

Demographic representativeness is a second limitation. The validation cohorts behind both assays, including the multicenter prospective 31-GEP cohort [23], the i31-GEP development series [8], the MERLIN_001 trial [12], and the independent Dutch validation [26], have been predominantly composed of non-Hispanic White patients, with Black, Hispanic, and Asian patients underrepresented or unreported. CM in patients of color, while less common, is typically diagnosed at a more advanced stage and follows a distinct anatomic distribution (acral, mucosal, subungual). Whether 31-GEP class or CP-GEP risk thresholds developed in White-predominant cohorts retain their prognostic accuracy in these populations is essentially uncharacterized. Selection bias of a different kind also operates within published cohorts: patients undergoing GEP testing in real-world practice are systematically more likely to be insured, geographically proximate to academic centers, and engaged with surveillance, all of which independently affect outcomes.

Survey data suggest patients desire additional prognostic information [32] and that GEP are already in use guiding clinical decision-making and referral patterns in the community. It is tempting to view GEP results as catch-all values offering clear guidance on SLNB, however a close look at the published data reveals that their usefulness depends on context, and the user’s understanding of both their patient and the test.

## 7. Future Directions

Several areas warrant further attention. First, the approval of pembrolizumab as adjuvant therapy for resected stage IIB/IIC melanoma and of nivolumab in completely resected disease introduces a new dimension to risk stratification [31]. GEP assays may help identify which stage II patients are most likely to benefit from adjuvant immunotherapy, potentially sparing low-risk patients from toxicity while ensuring high-risk patients are treated appropriately. However, no peer-reviewed studies to date have demonstrated GEP-class prediction of response or benefit to adjuvant therapy in melanoma, and incorporation into treatment selection algorithms is not currently supported.

Second, integration of GEP with liquid biopsy, particularly ctDNA, is a promising frontier. Like GEP, ctDNA assays are clinically available and have demonstrated very high specificity, including in melanoma. Two situations are of particular interest for the patient being assessed for low vs. no residual melanoma. For the patient where it appears all visible disease would be resectable, such as before a sentinel lymph node biopsy, a non-negative ctDNA would have higher chance of more disease, favoring upstaging [33]. For the patient for whom all visible disease appears to have been resected, the ctDNA positive patients have recurrence at very high specificity [34,35]. Serial ctDNA testing for those initially negative can be used as a tipping point for radiologic testing or systemic treatment start, although there are some recurrences still among ctDNA negative patients.

A practical plan to coordinate a GEP assay with ctDNA in some scenarios could be organized thus (Table 3), although future study should organize the combinations more qualitatively.

Third, GEP should be situated within the broader trajectory of precision pathology. Current assays use targeted RT-PCR of bulk tissue. Spatial transcriptomics and single-cell RNA-seq have outperformed bulk-tissue signatures in predicting checkpoint-inhibitor response in early proof-of-concept melanoma cohorts [36]. Current bulk-tissue GEP averages signal across heterogeneous cell populations and cannot resolve where in the TME a transcript is expressed. Spatial methods complement this by providing architectural context. Current GEP assays are best viewed as a clinically actionable, cost-feasible bridge between classical histopathology and emerging higher resolution methods.

Fourth, the field needs prospective randomized trials of GEP-guided management versus standard care. Long-term follow-up from MERLIN_001, including recurrence and survival endpoints, will be essential, as will cost-effectiveness analyses incorporating quality-adjusted life-years and downstream utilization. Combined-biomarker strategies, pairing GEP with ctDNA, multiplex immune signatures (PD-L1, TIL density), and potentially with stress-adaptive or AR-related markers, represent a logical next step but require prospective evaluation beyond retrospective synthesis. Ongoing clinical studies in melanoma GEP are listed in Table 4.

## 8. Conclusions

There is promising evidence supporting the use of GEP in select clinical scenarios, specifically with high-risk T1a patients where SLNB remains a debated topic. Despite their retrospective nature, many studies suggest a benefit of GEP for risk stratifying patients with respect to survival outcomes beyond current AJCC staging. While GEP assays have undergone prognostic validation, the data surrounding their clinical utility remains limited.

Both 31-GEP and CP-GEP assays have an estimated list price of around $7000. However, most commercial insurances cover the majority of this cost and there are several manufacturer assistance programs that can substantially reduce the patient’s out-of-pocket costs. Compared to SLNB-related costs as previously discussed, the use of GEP assays could prove to be cost effective particularly in those patients who might forego the procedure and associated anesthesia and facility fees based on risk stratification.

At this time, GEP assays should be viewed as adjunctive tools that can enhance shared decision-making and personalized treatment planning. They should not be used to replace current standard practices as their clinical utility and outcome benefits remain incompletely validated at this time. As additional prospective trials complete enrollment and report outcomes, the role of GEP in melanoma management will continue to be refined.

## Figures and Tables

**Table 1:** Comparison of DecisionDx-Melanoma (31-GEP) and Merlin (CP-GEP) assays.

Feature	DecisionDx-Melanoma (31-GEP)	Merlin CP-GEP
Data analyzed	28 discriminating + 3 control genes + clinicopathologic variables (in i31-GEP)	8 genes + clinicopathologic variables
Method	RT-qPCR on FFPE tissue	RT-qPCR on FFPE tissue
Risk classification	Class 1A, 1B, 2A, 2B; continuous % (i31)	Binary high risk/low risk
Clinical outputs	SLN positivity risk + recurrence/metastasis risk	SLN metastasis risk

**Table 2:** Practical clinical scenarios for GEP application in cutaneous melanoma.

Clinical Scenario	Standard Staging Implication	Role of GEP	Evidence Base
Low-risk T1a, age > 65, no high-risk features	SLNB not recommended (<5% predicted positivity)	Not indicated; unlikely to change management	AJCC 8; Vetto JT 2019 [21]
High-risk T1a (Breslow > 0.5 mm with age < 42, head/neck site, LVI, or mitoses ≥ 2/mm^2^)	5–10% predicted positivity; SLNB discussed but not mandated	Strongest current evidence-based role; reclassifies majority into actionable <5% or >10% categories	Whitman ED 2021 [22]; Hieken TJ 2025 (MERLIN_001) [12]; Shannon AB 2023 [20]
T1b, non-ulcerated, low-mitotic	SLNB offered; ~5% positive nationally	May identify CP-GEP low-risk subgroup with very low SLN positivity; investigational	Hieken TJ 2025 [12]; Zijlker LP 2026 [26]
T2 or higher	SLNB clearly indicated	Refines recurrence-risk counseling and surveillance intensity; should not be used to forgo SLNB	SSO consensus [7]; Jarell A 2022 [27]
Resected stage II with adjuvant immunotherapy decision	Pembrolizumab approved for stage IIB/IIC; nivolumab for stage IIB/IIC in EU	No peer-reviewed predictive data; should not drive adjuvant therapy decisions outside research	SSO consensus [7]
Post-resection surveillance planning	No consensus on intensity by stage	Hypothesis-generating role in stratifying intensity; survival benefit of intensified surveillance not established	DECIDE [23]; Bailey CN 2023 SEER [28]

**Table 3:** Proposed framework for coordinating GEP testing with ctDNA in selected clinical scenarios.

GEP Assessed Risk	ctDNA	SLNB Recommendation	Observation vs. Active Adjuvant Treatment
GEP-low	0	Defer	Observe
GEP-low	Positive	Reconsider SLNB	Anticipate treatment
GEP-high	(Either result)	Plan SLNB	Treatment depends on serial evaluation with scan data also
**SLN biopsy result**			
SLNB negative	0	(n/a)	Favors observation
SLNB positive	0	(n/a)	Consider observation
SLNB positive or negative	ctDNA initially or later positive	(n/a)	Anticipate active systemic treatment

**Table 4:** Ongoing clinical studies evaluating GEP assays in CM.

Feature	DECIDE (Beard 2026 [23] , Future Oncology)	MERLIN_001 (Hieken 2025 [12] , JAMA Surgery)	CORRESPOND (NCT06566404)
Assay	DecisionDx-Melanoma i31-GEP (Castle Biosciences)	Merlin CP-GEP	Merlin CP-GEP + ctDNA (SkylineDx)
Study Design	Prospective, multicenter observational registry; 30 US centers; May 2020–January 2025	Prospective, multicenter, blinded prognostic study; 9 US academic centers	Prospective, observational cohort; enrollment ongoing (~36 months anticipated)
Sample Size (N)	912 enrolled (431 SLN assessed; 482 SLNB not performed)	1761; all underwent SLNB per current guidelines	TBD; accrual ongoing
Target Population	T1–T4 CM; SLNB-eligible; non-metastatic	T1–T3 CM; SLNB-eligible per standard criteria	>pT3b CM; SLNB-eligible; non-metastatic; clinically node-negative
Primary Endpoint(s) and Key Results	Endpoints: confirm 31-GEP performance predicting SLN positivity; RFS in patients guided to avoid SLNB; impact on clinical SLNB management. Results: SLN positivity 2.6% in low-risk (<5%) predicted group, 1.8% in T1–T2a subgroup; 3-year RFS 97.8% in low-risk patients.	Endpoint: NPV for SLN positivity in CP-GEP low-risk group; SLN positivity by CP-GEP risk class. Results: CP-GEP low-risk patients had 7.1% SLNB positivity; high-risk 3-fold higher positivity; NPV 92.9%.	Endpoint: model performance for nodal positivity prediction. Results: no primary endpoint results published; accrual pending.

## Data Availability

No new data were created or analyzed in this study. Data sharing is not applicable to this article.
